# Children with *Plasmodium vivax* infection previously observed in Namibia, were Duffy negative and carried a c.136G > A mutation

**DOI:** 10.1186/s12879-021-06573-y

**Published:** 2021-08-21

**Authors:** Daniel Hosea Haiyambo, Larysa Aleksenko, Davies Mumbengegwi, Ronnie Bock, Petrina Uusiku, Benoit Malleret, Laurent Rénia, Isaac Kweku Quaye

**Affiliations:** 1 Department of Biochemistry and Microbiology, University of Namibia School of Medicine, Windhoek, Namibia; 2 Department of Clinical Sciences, University of Lund, Lund, Sweden; 3 Multidisciplinary Research Center, University of Namibia, Windhoek, Namibia; 4 Department of Biology, University of Namibia, Windhoek, Namibia; 5 Ministry of Health and Social Services Department of Biology, National Vector Borne Disease Control Program, Windhoek, Namibia; 6 Department of Microbiology and Immunology, Yong Loo Lin School of Medicine, National University of Singapore, Singapore, Singapore; 7 Immunology Translational Research Programme, Yong Loo Lin School of Medicine, National University of Singapore, Singapore, Singapore; 8 Immunology Programme, Life Sciences Institute, National University of Singapore, Singapore, Singapore; 9 Singapore Immunology Network (SIgN), Agency for Science, Technology and Research (A*STAR), Biopolis, Singapore; 10 A*STAR ID Labs, Agency for Science, Technology and Research (A*STAR), Biopolis, Singapore; 11 Lee Kong Chian School of Medicine, Nanyang Technological University, 11 Mandalay Road, Singapore, 308232 Singapore; 12 Faculty of Engineering, Computer and Applied Sciences, Regent University College of Science and Technology, Dansoman, P. O. Box DS 1636, Accra, Ghana

**Keywords:** Duffy gene mutations, Namibia, *Plasmodium vivax*

## Abstract

**Background:**

In a previous study, using a molecular approach, we reported the presence of* P. vivax* in Namibia. Here, we have extended our investigation to the Duffy antigen genetic profile of individuals of the same cohort with and without *Plasmodium* infections.

**Methods:**

Participants with *P. vivax *(*n* = 3),* P. falciparum* (n = 23) mono-infections and co-infections of *P. vivax/P. falciparum *(*n* = *4*), and *P. falciparum*/*P. ovale* (n = 3) were selected from seven regions. Participants with similar age but without any *Plasmodium* infections (n = 47) were also selected from all the regions. Duffy allelic profile was examined using standard PCR followed by sequencing of amplified products. Sequenced samples were also examined for the presence or absence of G125A mutation in codon 42, exon 2.

**Results:**

All individuals tested carried the − 67 T > C mutation. However, while all *P. vivax* infected participants carried the c.G125A mutation, 7/28 *P*. *falciparum* infected participants and 9/41 of uninfected participants did not have the c.G125A mutation. The exon 2 region surrounding codon 42, had a c.136G > A mutation that was present in all *P. vivax* infections. The odds ratio for lack of this mutation with *P. vivax* infections was (OR 0.015, 95% CI 0.001–0.176; p = 0.001).

**Conclusion:**

We conclude that *P. vivax* infections previously reported in Namibia, occurred in Duffy negative participants, carrying the G125A mutation in codon 42. The role of the additional mutation c.136 G > A in exon 2 in *P. vivax* infections, will require further investigations.

## Background

The life cycle of *Plasmodium* species in human hosts is initiated when an infected *Anopheles* mosquito injects sporozoites into the skin [1–3]. A fraction of the sporozoites move from the skin to circulation and take residence in the liver [4]. Here, the sporozoites multiply extensively to generate thousands of merozoites. Next, liver merozoites enter the circulation and infect red blood cells (RBCs) and initiate the erythrocytic cycle [5]. In RBCs, erythrocytic schizogony goes through a ring stage (immature trophozoites), mature trophozoites and merozoites stages, which is repeated overtime. This stage is responsible for the clinical symptoms of infection, due in part to RBCs lysis and release of their contents into the blood stream leading to a pro-inflammatory immune response [6]. *Plasmodium vivax *(*P. vivax*) is unique in that it has preference for immature reticulocytes during its erythrocytic cycle [7].

Duffy antigen receptor for chemokines (DARC) also called cluster of differentiation 234 (CD234) or atypical chemokine receptor (ACKR) is a receptor for a family of proinflammatory chemokines [8–10]. It was discovered to be a receptor for *P. knowlesi* and *P. vivax* and on the surface of RBCs in the 1970’s [11]. It is also present on the surface of endothelial cells [12]. The DARC glycoprotein is encoded by the *FY* gene. Two codominant alleles FY*A and FY*B exist. These alleles produce the respective antigens Fy^a^ and Fy^b^, which differ by a point mutation at position c.125G > A [13]. The Duffy negative/null antigen Fy^(a−b−)^ is associated with a cI-67 T > C mutation on the GATA-1 transcription factor binding motif of the Fy*A/B genes [14]. The mutation on Fy^b^ is commonly seen in sub-Saharan Africans, while the Fy^a^ mutation is common in Papua New Guineans [15, 16]. The null mutation on the Fy^b^ allele was thought to be responsible for resistance of invasion of reticulocytes by *P. vivax* in sub-Saharan Africans [17]. However, there have been cumulative evidence using microscopy and/or molecular tools for the presence of *P. vivax* parasites in Duffy negative individuals in Sub-Saharan Africans [18, 19]. Since most of these studies were performed with subjects who had mostly no travel history to known endemic areas, this raises the question as to whether *P. vivax* is now emerging, having adapted to invade reticulocytes independent of DARC. Alternatively, it is possible that *P. vivax* infection did always occur in Duffy negative individuals, but its diagnosis was overlooked.

As a first approach to assess the prevalence of *P. vivax* infection in Duffy negative infected people, active case detection needs to be done across sub-Saharan Africa. This will be the basis of the understanding of *P. vivax* bionomics allowing comprehensive studies of invasion of reticulocytes and survival in its human host.

We recently reported the identification of *P. vivax* in Namibia [20]. Here, we have followed up to assess the Duffy status of the subjects who were infected. Our results add to the growing evidence that *P. vivax* infected individuals can be Duffy negative.

## Methods

### Study sites and population selection

The details of the sample selection are as previously published [20]. Samples from the following regions respectively grouped into infected and uninfected, were included: Kunene (0:8), Omusati (3:8), Oshana (2:10), Ohangwena (7:5), Kavango West (2:2), Kavango East (17:7) and Zambezi (Caprivi) (2:7). *Plasmodium* infected participants previously published were selected and uninfected with similar ages were also selected. The *Plasmodium* infected participants totaled 33 while 47 were uninfected. The *Plasmodium* infected participants consisted of the following categories: *P. vivax* mono-infection (n = 3), *P. falciparum* mono-infection (n = 23), *P. vivax/P. falciparum* mixed infection (n = 4) and *P. falciparum/P. ovale* mixed infection (n = 3).

### Ethics statement

The study was approved by the Ministry of Health and Social Services (MOHSS) Ethical Committee, Namibia. All parents/guardians provided informed consent on behalf of all participants. Where necessary, assent was also obtained from the child before sample collection.

### Blood sample collection

The details of the procedure are as published previously [20]. In brief, an aliquot of 1.5–2.5 ml venous blood was collected into EDTA tubes and centrifuged at 3000 rpm for 5 min to separate the buffy coat, plasma and red blood cells into separate tubes. These were then stored at − 20 °C and later transferred to − 80 °C until analyzed.

### Laboratory analyses

#### DNA extraction

Genomic DNA was extracted from pelleted red blood cells using the automated Hamilton Star Microlab Workstation (Hamilton Bonaduz AG, Bonaduz, Switzerland) with the Machery and Nagel 96 blood DNA extraction kit. The starting blood sample was 200 μl of the packed and thawed red blood cells (RBCs), and final DNA elution volume of 120 μl sterile PCR-grade water.

#### Molecular detection of DARC

All detection assays were single-plexed and run in a high throughput 96 well plate Applied BioSystem GeneAmp 9700 PCR system, Singapore. Primers were ordered from Eurogentec, Liege, Belgium. All PCR amplification reactions were carried out in a total volume of 50 μl. For the standard PCR amplification reaction, 2.5 μl of DNA extracted from pelleted red blood cells was used, together with the KOD hotstart enzyme and the reaction mix was obtained from Sigma Chemical (Merck, Darmstadt, Germany). The amplified products were analyzed on 1.5% agarose gels by electrophoresis, followed by visualization on a UVP Geldoc-it Imager TS 310 (Cambridge, UK) after ethidium bromide staining. The amplified products were subsequently sent to Inqaba Biotech™, Pretoria, South Africa, for sequencing on both strands. Sequencing was done 2 × to confirm the observed mutation. The PCR cycling parameters for the primary amplifications were as follows: Initial denaturation at 95 °C for 2 min, then 30 cycles each of denaturation at 95 °C for 20 s, annealing at 62 °C for 10 s, extension at 70 °C for 11 s and a final hold at 4 °C. The primers had a final concentration of 0.2 µM with sequences of: Forward: CTCATTAGTCCTTGGCTCTTAC and Reverse: AGCTGCTTCCAGGTTGGCAC, and AGCTGCTTCCAGGTTGGCAT. The amplified products were 711 bp.

### Statistical analysis

Data were entered in an Excel data sheet and IBM Corp SPSS version 26 (IBM Corp. Released 2019. IBM Armonk, NY: USA) was used for analysis. Descriptive statistics and appropriate measures of central tendency were provided for relevant demographic covariates. Multinomial logistic regression was used to assess association of a mutation with a risk of infection with a *Plasmodium* parasite. A p value < 0.05 was considered significant.

## Results

**Fig. 1:**
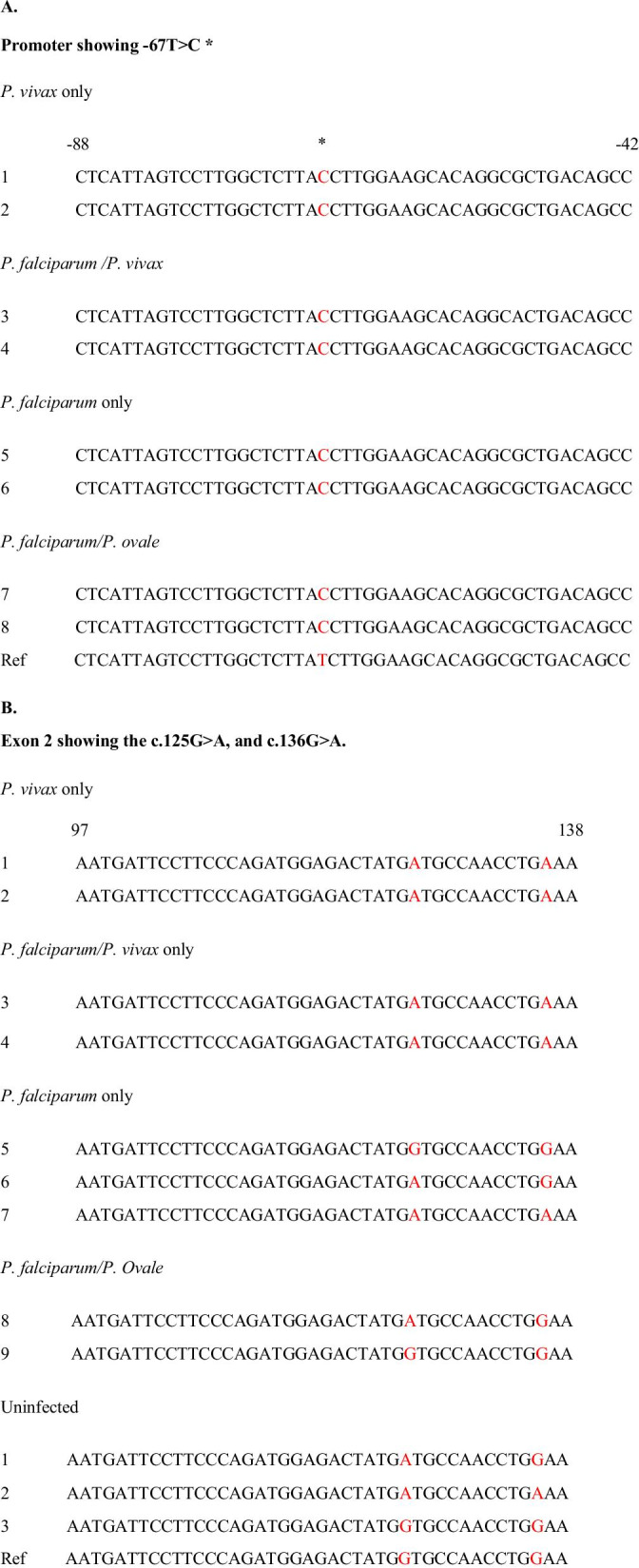
**A**, **B** Representative sequences in the ACKR gene of Plasmodium infected and uninfected participants with respect to the reference ACKR gene for − 67 T > C, c.125G > A and c.136G > A mutations

The median ages of the *Plasmodium* infected and uninfected individuals were respectively, 5 years (25–75 percentile, 2–8 years) and 6 years (25–75 percentile, 4–7 years). Data was obtained for all samples at the − 67 T > C locus. Similarly, data was obtained for *Plasmodium* infected participants at the c.125G > A and c136G > A locus. However, in uninfected participants, 41/47 sequence reads were obtained at the c.125G > A and c.136G > A locus (Table 1). *P. vivax* infected participants (5/7) were Duffy negative (− 67 T > C mutation) as were all other *Plasmodium* infected individuals tested (Fig. 1A). The remaining two *P. vivax* samples could not be examined because of insufficient DNA. All *P. vivax* infected participants had the c.125G > A mutation (Fig. 1B). However, 9/41 of the *Plasmodium* uninfected participants (6 samples reads were not good within the region) and 7/28 of the *P*. *falciparum* infected participants (Table 1), had the FY*A genotype (G at c.125 in exon 2) (Fig. 1B). There was a c.136 G > A mutation in exon 2 that was present in all *P. vivax* infections (5/7). The odds ratio for lack of the mutation with *P. vivax* infection was (OR 0.015, 95% CI 0.001–0.176; p = 0.001).

**Table 1:** Summary of the numbers of participants with mutations at − 67 T > C, c.125 G > A and c. 136 G > A for *Plasmodium* infected and uninfected participants

Mutations	*Plasmodium* infected				Uninfected
	*Pv*	*Pf/Pv*	*Pf*	*Pf/Po*	
− 67 T > C	3 (3)^a^	2 (2)	23 (23)	3 (3)	47 (47)
c.125G > A	3 (3)	2 (2)	17 (23)	2 (3)	32 (41)
c.136G > A	3 (3)	2 (2)	1 (23)	0 (3)	1 (41)

*Pv: P. vivax; Pf: P. falciparum; Po: P. ovale.*

^a^Numbers in bracket indicate the good sequence reads obtained

## Discussion

The present study compliments our previous investigation reporting the presence of *P. vivax* in Namibia [20]. The data clearly shows that the *P. vivax* infections previously observed in children, occurred in Duffy negative individuals. The observed mutation at c.136G > A occurring together with *P. vivax* Duffy negative infections in the FY*B allele needs to be examined further, as to whether it has any significant role in the infection dynamics of *P. vivax*. This mutation has not been previously published, to the best of our knowledge. Other rare DARC polymorphisms that have been reported are the c.265C > T mutation in the FY*B allele leading to the FY*X allele, which has a reduced expression of the gene by 90% [21], and the c.298G > A mutation resulting in a codon change from Ala100Thr [22], that reduces the expression of the Duffy antigen in erythrocytes. It is also interesting that none of the *P. vivax* infected participants carried a FY*A allele. *P. vivax* malaria is less benign than previously thought and considering that its life cycle is complicated by hypnozoites and early gametocyte release [23], the reports of the emergence of *P. vivax* in sub-Saharan Africa are a cause for concern. In Southern America it has been observed that recurrent infections of *P. vivax* frequently occur, even in subjects who had received full radical cure with primaquine [24, 25]. This shows that elimination of *P. vivax* is a difficult task requiring the National Malaria Control Programs (NMCPs) in African countries to plan accordingly.

The recent detections of *P. vivax* infections in Duffy-negative individuals suggest that the resistance associated with Duffy antigen negativity by *P. vivax* to reticulocyte infection is incomplete. [18]. The precise function of DARC on erythrocytes has not been deciphered. In general, however, it is known to be associated with the modulation of chemokine levels locally and systemically, to dampen inflammatory response [26, 27]. As to how this can impact parasite invasion and survival in the reticulocyte or erythrocyte is unknown. It has been reported that plasma and serum chemokine levels differ between individuals with FY*A and FY*B alleles [28]. The FY*B allele was observed to have a lower expression of the Duffy antigen than FY*A allele [29], although there was no effect on the binding affinities for chemokines. More data is required, examining new mutations in individuals infected or not infected with *Plasmodium* species to further understand the links with chemokine expression and inflammatory responses and how that relates to *P. vivax* entry into reticulocytes.

## Conclusion

The emergence of *P. vivax* infections in Duffy negative individuals in sub-Saharan Africa is a cause for concern. Namibia joins the list of these countries, which requires serious attention on the malaria elimination and eradication agenda of sub-Saharan Africa and partners.

## Data Availability

The datasets generated and/or analyzed during the current study are not publicly available due restrictions imposed by the Ethical Committee of the Ministry of Health and Social Services, but are available from the corresponding author on reasonable request.

## Abbreviations

RBCs: Red blood cells
DARC: Duffy antigen receptor for chemokines
CD234: Cluster of differentiation 234
ACKR: Atypical chemokine receptor
GATA: DNA binding sequence by transcription factors
FY: Duffy glycoprotein gene
MOHSS: Ministry of Health and Social Services
EDTA: Ethylene-diamine tetra acetic acid
PCR: Polymerase chain reaction
KOD: A DNA polymerase
UVP: Ultra violet protection
NMCP: National Malaria Control Program
